# Fatigue in brain tumor patients, towards a neuronal biomarker

**DOI:** 10.1016/j.nicl.2020.102406

**Published:** 2020-09-01

**Authors:** M.J. de Dreu, I.T. Schouwenaars, G.J.M. Rutten, N.F. Ramsey, J.M. Jansma

**Affiliations:** a Department of Neurosurgery, Elisabeth-Tweesteden Hospital, Tilburg, the Netherlands; b Department of Neurology and Neurosurgery, University Medical Center Utrecht Brain Center, Utrecht University, Utrecht, the Netherlands

**Keywords:** CEN, central executive network, DMN, default mode network, WMO, Dutch law for regulation of research involving human subjects, MG, meningioma, LGG, low grade glioma, HGG, high grade glioma, MFI-20, multidimensional fatigue inventory, MFI-total, a sum score of the MFI-20 ranging from 20 to 100, CUE, trial with an attention cue (green dot), CUE-STIM, trial with an attention cue (green dot) followed by a stimulus, ROI, region of interest, GDL, gnu data language, Fatigue, Brain tumor, fMRI, Biomarker, Phasic alertness

## Abstract

**Background:**

Many brain tumor patients suffer from fatigue which severely affects their quality of life. There is a lack of objective measurements for fatigue in brain tumor patients. We aimed to find a neurophysiological correlate for fatigue in brain tumor patients. For this purpose, we correlated brain activity associated with phasic alertness with self-reported ratings of fatigue.

**Methods:**

Patients with a meningioma, a low-grade glioma or a high-grade glioma (N = 63) participated in this fMRI study. Brain activity in the central executive network (CEN) and default mode network (DMN) associated with phasic alertness was correlated with self-reported fatigue measured with the multidimensional fatigue inventory (MFI-20). Follow-up analyses were performed for MFI-20 domain scores, individual regions within CEN and DMN, and the tumor sub-groups separately.

**Results:**

MFI-20 scores correlated significantly with DMN activity associated with phasic alertness, but not with CEN activity. These results were consistent for each tumor sub-group. Within the DMN, the correlations were strongest in left and right lingual cortex, left and right cuneus, and right precuneus.

**Discussion:**

Self-reported fatigue in brain tumor patients was associated with objective measurements of brain activity, specifically the DMN activity related to phasic alertness. This association represents an important step in the development of a biomarker for fatigue in brain tumor patients, and possibly for other patients that suffer from fatigue.

## Highlights

• Subjective fatigue correlated with phasic alertness default mode activation in brain tumor patients.
• Subjective fatigue did not correlate with phasic alertness central executive activation in brain tumor patients.
• Default mode activation is a candidate for developing a biomarker for fatigue in brain tumor patients.

## Graphical abstract

**Figure:**
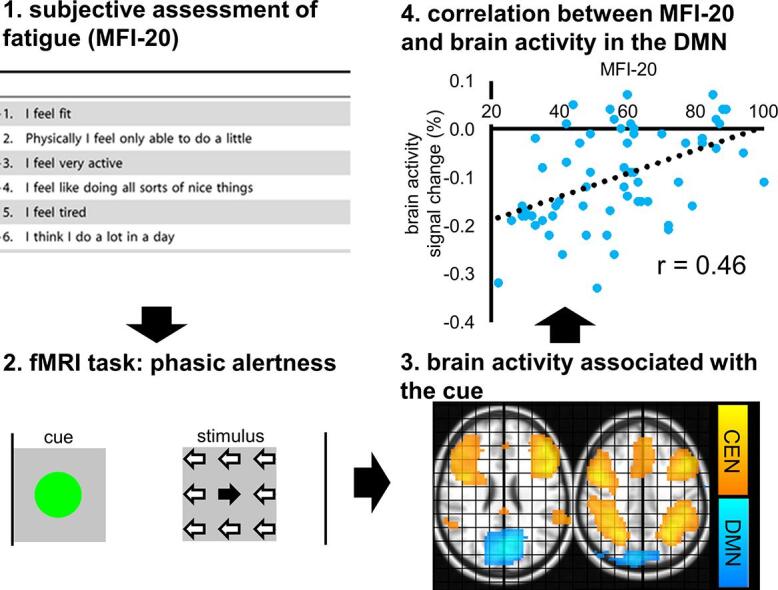

## 1 Introduction

Fatigue can be defined as a state where it is difficult to initiate and sustain voluntary activities (Chaudhuri and Behan, 2004), to process sensory information (Diamond et al., 2008), and to be alert or vigilant (Hanken et al., 2015). Fatigue is considered normal and healthy if it occurs after substantial physical or mental activity. However, it may be abnormal and a symptom of a disease if it occurs with insufficient provocation. In this paper we use the term fatigue specifically to refer to this pathological form.

In brain tumor patients, fatigue is a common symptom (Rooney et al., 2014, Valko et al., 2015, Armstrong et al., 2016, van Coevorden-van Loon et al., 2017, Linden et al., 2019) that has detrimental effects on quality of life (Cheng et al., 2010) and survival (Brown et al., 2008). The percentage of patients with a meningioma (MG) that experienced high levels of fatigue pre-surgery varied between 34% and 43% depending on which domain score was used (Linden et al., 2019). An astounding 82% of low-grade glioma patients (LGG) and 34% of high-grade glioma patients (HGG) reported pre-surgery fatigue (Cheng et al., 2010, Armstrong et al., 2016). These results show that fatigue is a common symptom for each of these three types of brain tumors.

Despite the high prevalence and severe consequences of fatigue in brain tumor patients as well as in other neurological and oncological patients (DeLuca et al., 2009, Berger et al., 2015, Penner and Paul, 2017), the pathophysiology is still largely unknown (Watt et al., 2000, DeLuca et al., 2009, Berger et al., 2015, Penner and Paul, 2017).

This lack of knowledge might be caused by a shortage of objective methods to measure fatigue. Although acute effects of fatigue can be measured objectively, also referred to as ‘fatigability’. Fatigability consistently fails to correlate with subjective fatigue and therefore self-reported fatigue and fatigability may represent different constructs (Kluger et al., 2013). Only in the alertness and vigilance domain task, performance measures have been associated with subjective fatigue (Hanken et al., 2015). Therefore, studying the neuronal correlate of a task from this domain might be a promising strategy to study fatigue.

Fatigue is mostly measured by self-report which is easily available, easy to use, and low in cost. However, self-report measurements are susceptible to interpretation differences, construct contamination (DeLuca et al., 2009), response shift, and recall bias (Visser et al., 2000, Althubaiti, 2016), which potentially reduces the reliability.

As was stated by Hanken et al. (2015), “If subjective fatigue is a feeling such as anxiety or pain, one would expect this feeling to be represented cortically”. A neurophysiological representation of fatigue measured with fMRI could provide a basis for an objective measurement. DeLuca et al. (2009) provided an extensive overview of imaging studies on fatigue. They concluded that the reviewed fMRI studies generally supported the hypothesis that fatigue is associated with damage to the cortical-subcortical circuitry. In brain tumor patients, this damage could be the result of brain displacement due to a growing tumor. More recently, resting state studies in multiple sclerosis patients suggested a possible involvement of the DMN in fatigue, as fatigue was associated with increased DMN connectivity, (Bisecco et al., 2018, Hogestol et al., 2019).

The aim of the current study is to find a neuronal correlate for fatigue in brain tumor patients. We included brain tumor patients with a low grade- or high grade glioma, or with a meningioma, since each of these three patient groups suffer from fatigue (Cheng et al., 2010, Armstrong et al., 2016, Linden et al., 2019). An objective neuronal correlate of fatigue in brain tumor patients could not only help increase the reliability of fatigue assessment, but also provide a new tool to study the pathophysiology of fatigue. For this aim, we focused on one of the most important aspects of fatigue, namely its detrimental effect on alertness (Hanken et al., 2015). In order to test if brain activity associated with phasic alertness also reflects fatigue, we correlated the phasic alertness brain response in both activated as well as deactivated regions (De Dreu et al., 2019) with self-reported fatigue ratings measured with the Multidimensional Fatigue Inventory (MFI-20) (Smets et al., 1995, Smets et al., 1996).

## 2 Methods

### 2.1 Participants

This study was carried out in accordance with the Medical Research Involving Human Subjects Act (WMO), and was approved by the Medical Research Ethics Committee Brabant [protocol number: NL51147.028.14]. All subjects gave written informed consent in accordance with the Declaration of Helsinki (2013).

Subjects were included if they were scheduled for surgical resection of an MG, LGG, or an HGG at the Elizabeth Tweesteden Hospital between July 2016 and February 2019. Clinical diagnosis was confirmed by histopathology after the surgery. Subjects were excluded if they reported a history of significant neurological (other than the tumor) or psychiatric disorder, claustrophobia, or if the subject was deemed not physically and mentally fit to complete testing by a neurosurgeon or specialized nurse.

The results in this study are based on 63 subjects (see Table 1 for patient characteristics). FMRI as well as MFI-20 measurements typically took place on the day of their admission to the hospital, which was generally one to five days before surgery.

**Table 1:** Socio-demographical and clinical characteristics.

	MG	LGG	HGG
N	23	21	19
Age (years),	54.3 (2.4)	38.7 (2.7)	54.5 (2.5)
Sex (male/female)	5/18	13/8	11/8
Education (Verhage), median (range)	5 (2–7)	5 (3–7)	5 (3–7)
Education (years)	15 (0.9)	15 (0.8)	15 (0.8)
Handedness (left/right)	1/22	2/19	3/16
Histopathological diagnosis			
WHO grade 1	22	2^a^	
WHO grade 2	1	19	1
IDH1+ astrocytoma		12	
IDH1+ oligodendroglioma		7	
IDH−			1
WHO-grade 3			1
IDH+			1
IDH−			
WHO-grade 4			17
Tumor hemisphere (left/right)	16/7	9/12	10/9
Tumor volume (cm^3^)	46.5 (6.4)	57.0 (11.5)	68.0 (13.1)
Tumor overlap CEN (%)	2.5 (0.7)	3.0 (1.1)	2.2 (1.1)
Tumor overlap DMN (%)	0.8 (0.6)	0.4 (0.2)	0.3 (0.1)

Variables are presented as mean (SEM) unless indicated otherwise. a = dysembryoplastic neuroepithelial tumor (DNET), abbreviations: MG = meningioma, LGG = low grade glioma, HGG = high grade glioma.

N = number of subjects, SEM = standard error of the mean, CEN = central executive network, DMN = default mode network.

### 2.2 Subjective fatigue

The MFI-20 is a widely used multidimensional questionnaire with adequate psychometric quality (Agasi-Idenburg et al., 2010). It has been developed specifically for measuring fatigue related to cancer (Smets et al., 1995) and has been recommended for use with brain tumor patients (Asher et al., 2016). Subjects completed the questionnaire at their own pace and time and returned it to the healthcare staff. The MFI-20 includes five domain scores (general fatigue, physical fatigue, reduced activity, reduced motivation, and mental fatigue). For this study, we additionally used a sum score of the entire MFI-20 questionnaire (MFI-total), as we expect that this would most likely reflect a general representation of fatigue, as well as maximize the spread in fatigue scores within our patient group.

### 2.3 Functional MRI

#### 2.3.1 Task design

For the fMRI experiment, we used a paradigm designed to isolate brain activity associated with phasic alertness that was based on (Shulman et al., 1999). Phasic alertness paradigms are typically used to study the effect of phasic alertness on an associated task. In this study, we focused specifically on the brain activity associated with phasic alertness itself, since we hypothesize that activation associated with phasic alertness is related to fatigue.

**Fig. 1:**
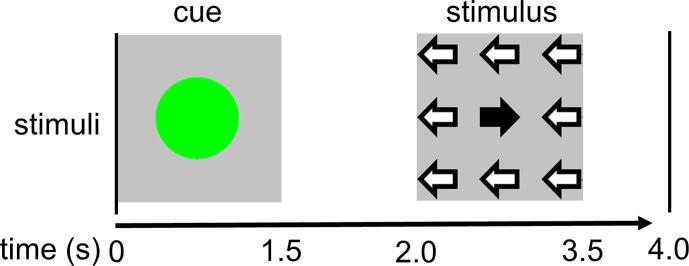
Representation of the visual stimuli. The cue consisted of a green dot. The stimulus of 9 black or white left and right pointing arrows in a 1 to 8 ratio. Each trial was 4 s. In 50% of the trials the cue was not followed by a stimulus. These trials were used for the fMRI analysis.

The task is outlined in Fig. 1: The task stimulus consisted of nine arrows in a three by three layout. Eight arrows pointed in one direction, one arrow in the opposite direction. Subjects were instructed to indicate the direction of the majority of the arrows, using their right index finger for answering ‘left’ and their right middle finger for answering ‘right’ on a pneumatic button box. The cue consisted of a green dot. Subjects were instructed to mentally prepare for an upcoming task stimulus if a cue was presented. In 50% of the trials, a stimulus followed the cue. The resulting two trial types will be denoted in this paper as: ‘CUE’ (a green dot not followed by a task-stimulus) and ‘CUE-STIM (a green dot followed by a task-stimulus). Both trial types were randomized to optimize GLM regressor independence between trial types and to prevent task predictability. Stimuli were presented by “Presentation” software (version 20.1, http://www.neurobs.com).

The total duration of the task was 9.2 min with four runs of 112 s, enveloped by 5 rest periods of 20 s consisting of a stationary black screen with the text: “you have a 30 s break” in Dutch and in white letters. These 5 rest periods were used as baseline. Both CUE and CUE-STIM occurred 20 times in each run. The duration of each trial was 4000 ms. Cues were presented at t = 0, with a duration of 1500 ms and stimuli were presented at t = 2000, also with a duration of 1500 ms. Subjects practiced the task outside the scanner following a standard practice protocol of approximately 5 min of explanation and a minimum of 4 min practice which was prolonged if the experimenter deemed it useful for the understanding of the task.

#### 2.3.2 Scan session

The total duration of the scanner session differed slightly between subjects because clinical and research scans were combined. In each session, the task started after approximately 10 min of scanning.

#### 2.3.3 Image acquisition

Scans were performed on a 3T Philips Achieva scanner (Philips Medical Systems, Best, the Netherlands) using a 32-channel SENSE head coil. A 3D T1-weighted structural image was acquired for anatomical registration purposes (scan parameters: TR/TE: 8.4/3.8 ms, FOV: 254 × 254 × 158 mm3, flip angle: 8°, voxel size 1 mm isotropic, 158 slices (sagittal orientation)). T1-contrast scans and FLAIR scans were used for tumor segmentation which enabled determination of tumor volume, tumor location, and tumor overlap with regions of interest (ROIs). FMRI images were obtained using an EPI pulse sequence (scan parameters: volume acquisition time 2 s, TR/TE: 2000/27.6 ms, FOV: 240 × 240 × 110, 7 mm, flip angle: 70°, voxel size 3 mm isotropic, 37 slices (transverse orientation), 278 volumes). A mirror attached to the head coil enabled subjects to see an opaque projection screen positioned behind the scanner. A video projector inside the scanner room projected the task on the screen.

### 2.4 Analysis

#### 2.4.1 Performance

Accuracy was calculated as the percentage of correct responses. Reaction time was calculated as the response time minus the stimulus presentation time for correct responses.

#### 2.4.2 FMRI preprocessing

FMRI data were preprocessed using SPM12 (Welcome Trust Centre for Neuroimaging, University College London, London, UK: http://www.fil.ion.ucl.ac.uk/spm/software/spm12/)*.* Scans from the session were realigned to the first scan to correct for subject movement using a least squares approach, a 6 parameter (rigid body) spatial transformation, and a 2nd degree B-spline interpolation. The scans were co-registered to the T1 using a rigid body model. The parameters were estimated by a normalized mutual information function. The images were resliced by a 4th degree B-spline. The T1 was spatially normalized into standard MNI-space using very light bias regularization (0.0001) and a 4th degree B-Spline. The resulting parameters were applied to all functional scans in order to account for anatomical differences and therefore enable group analysis. Finally, all scans were spatially smoothed with a 3D Gaussian filter (full-width at half-maximum: 12 mm) to further minimize the effect of functional anatomical differences.

For the evaluation of the fMRI data, a single subject event-related GLM regression analysis was performed with two regressors of interest, modelling the expected BOLD signal associated with CUE and CUE-STIM trials, using the canonical BOLD model included in SPM12 without derivatives. SPM applied the GLM to estimate the correlation between the task regressor and the BOLD response, hereafter called 'the signal'. Additionally, regressors functioned as a high pass filter with a 128 s cut-off. Beta coefficients were transformed to represent the percent signal change for each voxel. Percent signal changes were calculated for CUE as well as CUE-STIM trial types.

#### 2.4.3 ROI selection

ROIs were based on a full-brain systematic grid that defined 628 cubic ROIs of 15 by 15 by 15 mm (3375 mm^3^). This grid was specifically designed for fMRI group analysis to enable flexible, reproducible, as well as simple ROI selection. Each ROI included a maximum of 125 3 mm isotropic voxels. For each ROI, an average activation level was calculated using all voxels. Although using predefined systematic ROIs results in a reduced spatial match with activation peaks compared to regular ROI definition (Poldrack 2007), this disadvantage is outweighed by the benefits, as it removes any risk of circular analysis (Kriegeskorte et al., 2009), and facilitates quantitative comparison of fMRI results within studies, between ROIs, conditions or groups, as well as between studies (Jansma and Rutten, 2017, De Dreu et al., 2019, Schouwenaars et al., 2019, Schouwenaars et al., 2020).

**Fig. 2:**
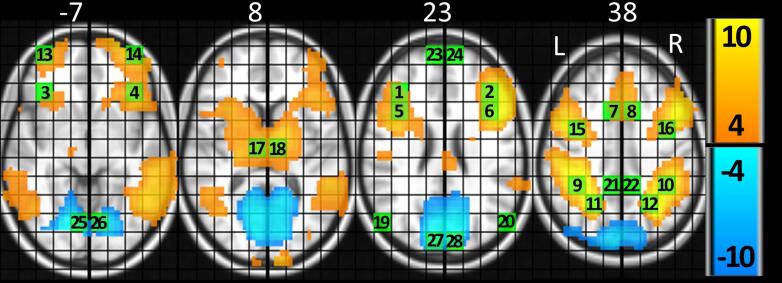
Overview of whole brain fMRI results and ROI location for all patients. T-values for CUE versus baseline (≥4 = orange, ≤−4 = blue, see Table 2 for the names and abbreviations of the ROIs. MNI coordinates are provided above each slice. Abbreviations: L = left, R = right. (For interpretation of the references to colour in this figure legend, the reader is referred to the web version of this article.)

ROIs were selected via visual inspection of activation hotspots in the CUE group activation map. If the activation map showed a significant signal change in only one hemisphere, the mirror ROI in the other hemisphere was also included in order to increase the chance to include all regions with an involvement in phasic alertness (Fig. 2, Table 2).

**Table 2:** ROI characteristics.

a									
		BA	MNI			Task-baseline contrast			Tumor overlap (%)
NW	ROI number and name (ACRO)		x	y	z	mean (SEM)	T	p	
CEN	1 L dorsolateral prefrontal cortex (LDLPFC)	48	−39	30	30	0.09 (0.01)	6.62	<0.001	0.2
	2 R dorsolateral prefrontal cortex (RDLPFC)	48	39	30	30	0.08 (0.01)	5.07	<0.001	3.5
	3 L ventrolateral prefrontal cortex (LVLPFC)	47	−39	30	0	0.06 (0.01)	5.95	<0.001	1.6
	4 R ventrolateral prefrontal cortex (RVLPFC)	47	39	30	0	0.04 (0.01)	3.15	0.001	3.9
	5 L premotor cortex (LPC)	44	−39	15	30	0.11 (0.01)	7.60	<0.001	1.6
	6 R premotor cortex (RPC)	48	39	15	30	0.08 (0.01)	6.24	<0.001	0.0
	7 L anterior cingulate cortex (LACC)	32	−9	15	45	0.10 (0.01)	7.88	<0.001	1.4
	8 R anterior cingulate cortex (RACC)	32	9	15	45	0.06 (0.01)	6.14	<0.001	2.3
	9 L Inferior Parietal cortex (LIPC)	40	−39	−45	45	0.15 (0.01)	11.98	<0.001	4.6
	10 R Inferior Parietal cortex (RIPC)	40	39	−45	45	0.18 (0.01)	13.38	<0.001	4.9
	11 L Superior Parietal cortex (LSPC)	7	−24	−60	45	0.11 (0.01)	10.68	<0.001	6.2
	12 R Superior Parietal cortex (RSPC)	7	24	−60	45	0.13 (0.01)	11.21	<0.001	4.7
	13 L Middle frontal gyrus, op (LMFGop)	0	−39	60	0	0.18 (0.03)	6.75	<0.001	3.0
	14 R Middle frontal gyrus, op (RMFGop)	10	39	60	0	0.18 (0.05)	3.93	<0.001	1.1
	15 L Precentral gyrus (LPCG)	6	−39	0	45	0.10 (0.01)	8.45	<0.001	0.0
	16 R Precentral gyrus (RPCG)	6	39	0	45	0.11 (0.01)	8.75	<0.001	7.1
	17 L Thalamus (LTHAL)	0	−9	−15	15	0.08 (0.02)	5.02	<0.001	0.0
	18 R Thalamus (RTHAL)	0	9	−15	15	0.07 (0.01)	5.45	<0.001	0.0

b									

DMN	19 L Angular Gyrus (LAG)	0	−54	−75	30	−0.03 (0.02)	1.73	0.044	0.0
	20 R Angular Gyrus (RAG)	0	54	−75	30	−0.02 (0.02)	1.31	0.098^a^	0.8
	21 L precuneus (LPCUN)	0	−9	−45	45	−0.04 (0.01)	3.19	0.001	1.3
	22 R Precuneus (RPCUN)	0	9	−45	45	−0.02 (0.01)	2.15	0.018	0.3
	23 L medial prefrontal cortex (LMPC)	10	−9	60	30	−0.02 (0.02)	0.75	0.227^a^	1.6
	24 R medial prefrontal cortex (RMPC)	10	9	60	30	−0.05 (0.02)	2.17	0.017	0.5
	25 L Lingual cortex (LLING)	18	−9	−75	0	−0.21 (0.03)	7.92	<0.001	0.0
	26 R Lingual cortex (RLING)	17	9	−75	0	−0.20 (0.02)	9.36	<0.001	0.0
	27 L Cuneus (LCUN)	18	−9	−90	30	−0.19 (0.02)	7.81	<0.001	0.4
	28 R Cuneus (RCUN)	0	9	−90	30	−0.20 (0.02)	8.18	<0.001	0.0

Abbreviations: ROI = region of interest, MNI = Montreal neurological institute, NW = network, ACRO = acronym, BA = Brodmann area, NV = number of voxels, SEM = standard error of the mean, L = left, R = right, CEN = central executive network, op = orbital part, DMN = default mode network. ^a^ ROIs with low (insignificant) activity remained in the analysis because their left or right counterpart showed significant activity

ROIs with increased activation compared to baseline were allocated to the central executive network (CEN). ROIs with a decreased activation compared to baseline were allocated to the default mode network (DMN). For network analysis, the average percent signal change per network per subject was calculated.

It can be expected that the fMRI signal is reduced if an ROI overlaps with a brain tumor (Larjavaara et al., 2007, Hirayama et al., 2018). To address this issue, the number of voxels with tumor overlap was calculated per network, per ROI, and per subject using GNU Data Language (GDL; (Arabas et al., 2010)). *Tumor overlap per ROI* was calculated as: ∑ *number of voxels with tumor overlap* divided by (∑ *total number of voxels in fMRI analysis* multiplied by *number of subjects*) multiplied by 100% (Table 2). *Tumor overlap per subject* was calculated as: ∑ *number of voxels with tumor overlap* divided by (*total number of voxels in fMRI analysis* multiplied by *number of voxels for this ROI*) multiplied by 100%. Additionally, the percent overlap was correlated with MFI-total scores. This evaluation indicated that the overlap of an ROI with a tumor in any ROI was negligible (see results section and Table 1, Table 2). Therefore, this variable was not further taken into account.

#### 2.4.4 FMRI group analysis

**Fig. 3:**
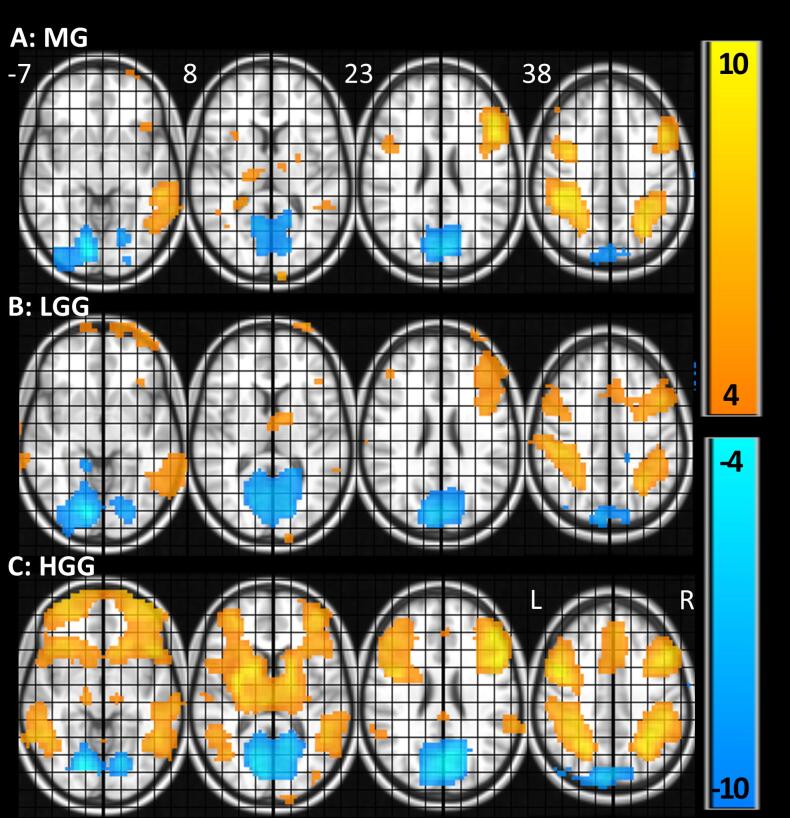
Overview of whole brain fMRI results for MG, LGG, and HGG separately. The activity pattern for each sub-group similar. T-values for CUE versus baseline (≥4 = orange, ≤−4 = blue. MNI coordinates are provided above each slice. Abbreviations: MG = meningioma, LGG = low grade glioma, HGG = high grade glioma, L = left, R = right. (For interpretation of the references to colour in this figure legend, the reader is referred to the web version of this article.)

The individual activation maps were used to perform a network analysis executed with GNU data language (GDL). A second level voxel-wise group analysis was performed for visualization of group activation patterns for all patients combined (Fig. 2) and for MG, LGG, and HGG separately (Fig. 3).

#### 2.4.5 Statistical analysis

The data resulting from MFI-20 is generally treated as interval data (Watt et al., 2000). However, because it stems from ordinal data, Spearman’s correlation analysis was used to test our two main hypotheses, namely the correlation between MFI-total and signal change in CEN and DMN. Follow-up analyses included correlation analyses for each separate MFI-domain score and correlation analyses for MFI-total per patient subgroup. To evaluate the contribution of each ROI that was included in the CEN and DMN network, a correlation analysis was performed between MFI-total and signal change in each ROI of CEN and DMN separately.

Because we applied two main hypothesis tests, these results were corrected for multiple comparisons using a Bonferroni correction. If this main hypothesis test was significant, follow-up tests were interpreted as a further evaluation of a significant main result and not corrected for multiple comparisons. If the main hypothesis test was not significant, follow-up tests were interpreted as post-hoc hypothesis tests, and also corrected for multiple comparisons.

**Fig. 4:**
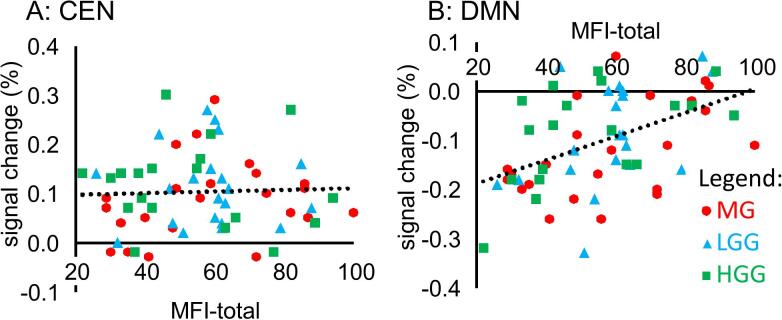
Overview of the linear relation between percent signal change of the DMN and CEN with MFI-total. MG patients are represented in red circles, LGG patients are represented in blue triangles, HGG patients are represented in green squares. Abbreviations: DMN = default mode network, MFI-total = sum score of fatigue questionnaire, MG = meningioma patients, LLG = low grade glioma, HGG = high grade glioma. (For interpretation of the references to colour in this figure legend, the reader is referred to the web version of this article.)

**Fig. 5:**
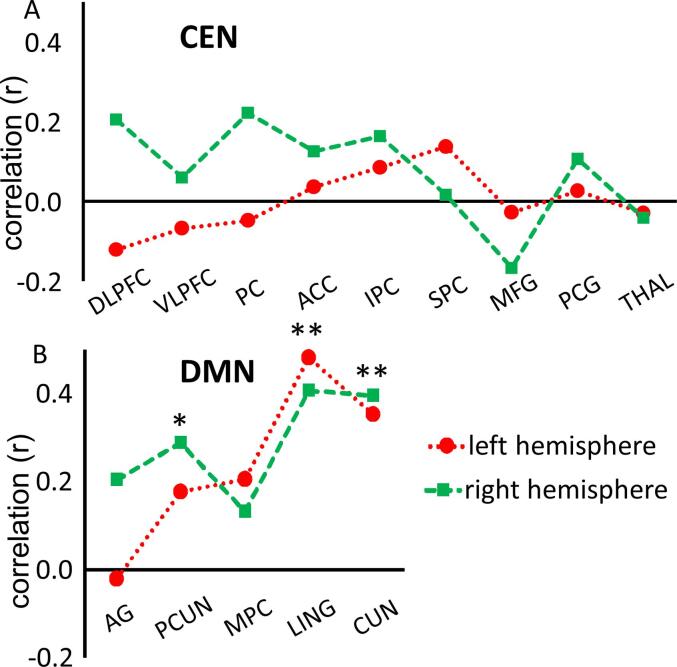
Overview of exploratory analysis of correlation strength (r) between signal change and MFI-total score for each ROI separately. * Correlation is significant at p < 0.05 for left precuneus, ** Correlation is significant at p < 0.001 for left and right lingual cortex and left and right cuneus. Abreviations: CEN = central exectutive network, DLPFC = dorsolateral prefrontal cortex, VLPFC = ventrolateral prefrontal cortex, PC = premotor cortex, ACC = anterior cingulate cortex, IPC = inferior parietal cortex, SPC = superior parietal cortex, MFG = middle frontal gyrus, PCG = precentral gyrus, THAL = thalamus, AG = angular gyrus, PCUN = precuneus, MPC = medial prefrontal cortex, LING = lingual cortex, CUN = cuneus

IBM SPSS statistics version 23 was used for statistical analysis. MRIcron was used for Fig. 2, Fig. 3, and Excel was used for Fig. 4, Fig. 5.

## 3 Results

### 3.1 Demographic and clinical characteristics

The demographic and clinical subject characteristics are summarized in Table 1. As expected, the LGG patients are relatively young compared to the MG- and HGG patients, there is a relatively high number of female MG patients, and there are differences in histological pathology between the tumor subtypes (Table 1). Furthermore, all subjects complied with the task instruction, as evidenced by high task accuracy (mean ± SEM; 96.9% ± 0.9; response time: 900 ms ± 24). Tumor-ROI overlap was small, since it did not exceed 3.0% of voxels for any network (Table 1) and it did not exceed 7.1% for any ROI (Table 2). More important, there was no correlation between the tumor overlap per subject and the MFI-total score (r (61) = −0.08, p = 0.55), which suggests that the influence of tumor overlap on the main study results is small. Fig. 3 displays the voxel-wise activity pattern for MG, LGG, and HGG separately. The activity patterns for each sub-group are similar.

### 3.2 Correlation analysis

#### 3.2.1 Network results

Our network correlation analysis indicated that MFI-total did not correlate significantly with the signal change in the CEN during CUE (r (61) = 0.001, p = 0.995; Fig. 4A; Table 3).The MFI-total score did correlate significantly with the signal change in the DMN during CUE (r (61) = 0.47, p < 0.001; Fig. 4B; Table 3). This pattern of results was consistent for each MFI domain score (Table 3) and for the three tumor sub-groups. For each subgroup, the correlation between the MFI-total score and the signal change in the CEN was not significant (MG: r(21) = 0.22, p = 0.33, LGG: r(19) = −0.01, p = 0.97, HGG: r(17) = −0.1, p = 0.69 and for each subgroup the correlation between the MFI-total score and the signal change in the DMN was significant (MG: r(23) = 0.52, p = 0.01, LGG: r(21) = 0.48, p = 0.03, HGG: r(19) = 0.51, p = 0.03), which is consistent with the main results.

**Table 3:** Spearman’s correlation between fatigue and signal change at network level.

Domain	CEN		DMN	
	r	p	r	p
MFI total	0.001	0.995	**0.47**	<0.001
General fatigue	−0.09	0.47	**0.38**	0.002
Physical fatigue	−0.01	0.97	**0.41**	0.001
Reduced activity	0.06	0.62	**0.46**	<0.001
Reduced motivation	−0.02	0.86	**0.38**	0.002
Mental fatigue	0.15	0.25	**0.33**	0.009

Abbreviations: p = p-level.

#### 3.2.2 ROI results

The exploratory follow-up analyses showed a significant negative correlation between MFI-total and percent signal change for ROI 25 (left lingual cortex; r(61) = 0.48, p < 0.001), ROI 26 (right lingual cortex; r(61) = 0.41, p = 0.001), ROI 27 (left cuneus; r(61) = 0.35, p = 0.005), ROI 28 (right cuneus; r(61) = 0.40, p = 0.001), and ROI 22 (right precuneus; r(61) = 029, p = 0.02; Fig. 5). All ROIs showing a significant correlation with MFI-total were part of the DMN.

## 4 Discussion

### 4.1 Current findings

The objective of this study was to find a neuronal correlate for fatigue in brain tumor patients. Our study results suggest that there are indeed signals in the brain that reflect the self-reported feeling of fatigue. Specifically, the level of self-reported fatigue was significantly correlated with the level of DMN deactivation associated with phasic alertness. In contrast, self-reported fatigue did not correlate with CEN activation associated with phasic alertness. We believe that this finding is a promising step towards the development of an objective neuronal biomarker for fatigue in brain tumor patients and potentially for other patients. The association between fatigue and a reduced capacity to inhibit DMN activation may furthermore provide a potential new lead for studies on the pathophysiology of fatigue.

The correlation results were consistent for each domain of the MFI-20, which could be interpreted as indication of the robustness of our findings. Alternatively, it may also be an indication that the subdomains of the MFI-20 questionnaire have a certain level of dependence. The correlation results also were surprisingly consistent for each of the three patient groups (MG, LGG and HGG), despite clear differences in pathology between these diseases. This suggests that the neural correlate of fatigue presented in this paper might be generalized to other patient populations.

Within the DMN network, the strongest correlation was found in the left and right lingual cortex, left and right cuneus, and right precuneus, all of which are located in the visual cortex. Possibly, deactivation of these ROIs is directly related to visual concentration, which is an important factor in the visual perception task used in our experiment. This result also suggests that the domain of a task may affect the activation pattern of an associated warning cue, which may also explain why the posterior cingulate cortex showed no signal change in the current study, despite being an important region within the DMN.

### 4.2 Relevance

While measuring fatigue via self-report is sensitive to interpretation differences, response shift, and recall bias (Visser et al., 2000, Althubaiti, 2016), a neuronal biomarker for fatigue does not suffer from these weaknesses. Thus, the observed neuronal correlate represents an important first step in the development of a biomarker for fatigue. A neuronal biomarker for fatigue could be especially important in clinical or research settings where self-reported measurements of fatigue may lead to systematic errors. An example of such a systematic error is response shift, which results in patients rating their symptoms differently because of previous experiences. The risk of a response shift is highest when patients experience significant changes in their symptom severity between measurements (Andrykowski et al., 2009). Therefore, measuring fatigue through an objective neuronal biomarker would be especially useful in longitudinal studies that include patients who potentially experience severe levels of fatigue, for example patients undergoing chemo- and radiation therapy (Berger et al., 2015). A similar mechanism may also affect cross-sectional study designs because patients may have a higher chance to be influenced by recent experiences of severe fatigue than a control group. Therefore, these groups may have a different perspective on fatigue, which may impact self-reported measures of fatigue. In these situations, an fMRI-based biomarker of fatigue may improve reliability as well as validity of the measurement of fatigue. An fMRI biomarker of fatigue may thus be a potentially important complement to self-reported fatigue in studies and clinical settings.

It could be argued that an fMRI scan is too expensive for routine application as a tool to gather objective information about the level of fatigue in brain tumor patients. However, this measurement could be combined with a structural MRI as well as presurgical fMRI scans, which are often part of standard care for brain tumor patients which would strongly reduce costs. Perhaps most importantly, this fMRI task could be useful to study the neuronal mechanism of fatigue, for example by comparing the neuronal correlate of fatigue between patients suffering from fatigue in different disease stages or with different diseases.

### 4.3 Relationship with previous literature

Previous reviews on the topic of fatigue and neuroimaging have reported that fatigue is most likely associated with abnormal activity in frontal cortex and basal ganglia (Chaudhuri and Behan, 2004, DeLuca et al., 2009). However, these conclusions have been mostly based on studies defining fatigue as a change in activation or performance decrement over time (i.e. fatigability; (DeLuca et al., 2008, Kohl et al., 2009, Moller et al., 2017). Fatigability correlates poorly with self-reported fatigue, suggesting they are independent constructs (Kluger et al., 2013). Consequently, the neural correlate of fatigability may not be applicable for self-reported fatigue and fatigability studies may not explain why patients experience fatigue that interferes with their lives.

To our knowledge, there are no previous task-based fMRI studies that have focused on a possible association between the level of task-induced deactivation and fatigue in brain tumor patients, other diseases, or healthy controls. However, there are several fMRI studies using connectivity analysis that also implicate the DMN in fatigue. Shan et al. (2018a) reported decreased connectivity between the left inferior parietal lobve (corresponding to the angular gyrus, ROI 19) and the medial prefrontal cortex (corresponding to the left precuneus, ROI 23, and right medial prefrontal cortex, ROI 24). Shan et al. (2018a) reported that the temporal complexity of fMRI time series in the DMN correlated with health scores in chronic fatigue patients. Bisecco et al. (2018) concluded that fatigue was mainly driven by connectivity changes in the DMN, after studying resting-state functional connectivity of the DMN and sensorimotor network in relapsing remitting multiple sclerosis patients with and without fatigue using independent component analysis. A study by Hogestol et al. (2019) indicated that symptoms of fatigue were reflected in altered DMN connectivity. Higher DMN connectivity was seen in MS patients with fatigue even with low depression scores in patients with MS. Higher connectivity is generally interpreted to represent stronger connection between regions of a network. Possibly this stronger connectivity makes it a more stable network, which makes it more difficult to inhibit this network. It might be easier to implement resting state fMRI scans than task-based fMRI scans in clinical practice. Therefore, if measures based on connectivity analysis show similar correlation strength with self-reported fatigue, they could also be useful as biomarkers for fatigue.

### 4.4 DMN

Our finding that the deactivation of the DMN can occur prior to task execution is in line with several previous findings (Sidlauskaite et al., 2014, De Dreu et al., 2019). Previous studies have also shown that the DMN deactivates during a wide range of cognitive tasks (Shulman et al., 1997, Laird et al., 2009). It has also been shown that the level of deactivation is correlated with task difficulty (McKiernan et al., 2003, Jansma et al., 2007a, Jansma et al., 2007b, Singh and Fawcett, 2008, Mangina et al., 2009, Ceko et al., 2015). It has been hypothesized that inhibition of the DMN is a prerequisite for goal oriented behavior (Raichle et al., 2001). Failure to inhibit the DMN prior to a cognitive task has also been shown to increase with response latency (Weissman et al., 2006). Together with the current results, this suggests a possible mediating role of DMN between fatigue and cognitive performance that is directly associated with phasic alertness.

Despite an abundance of literature and interest in the DMN, the exact functions are still mostly unknown. Previous studies have speculated that the DMN is, involved in the regulation of homeostasis and the autonomic nervous system (Jansma et al., 2007a, Jansma et al., 2007b, Shmueli et al., 2007, Raichle, 2015, Iacovella et al., 2018). This interpretation fits well with our findings, as regulation of homeostasis could benefit recovery from disease.

### 4.5 CeEN

In the present study, self-reported fatigue did not correlate with CEN activation, which is generally associated with task execution. This is in contrast to the results reported by Filippi et al. (2002). Filippi et al. (2002) reported a negative correlation between self-reported fatigue and activation in the contralateral intraparietal sulcus, thalamus, and ipsilateral Rolandic operculum in multiple sclerosis patients. This interesting discrepancy may be associated with the fact that Filippi et al. (2002) associated fatigue with the task activity itself. This has the disadvantage that the results may also reflect differences in task performance. Alternatively, the different patient populations (multiple sclerosis patients versus brain tumor patients) may also have contributed to the different findings.

### 4.6 Limitations

The present study was designed to search for a neuropsychological correlate for fatigue in brain tumor patients. Any conclusions about a causal association between DMN and fatigue fall outside of the scope of this paper.

Our study only included brain tumor patients, so our results only apply to this population. However, there is no evidence that the role of the DMN in cognition in brain tumor patients differs from that in other patient populations. Furthermore, the results are remarkably consistent between the tumor sub-groups, despite demographical and pathological differences. Therefore, it is possible that similar results could be found in other patient populations that suffer from fatigue.

The effect of a cue on performance could not be evaluated in the current study because no trials were included where a stimulus was not preceded by a cue. The main reason for this choice was the fact that trials without a cue might have reduced the effect of the cue, because patients might be tempted to stay alert during the task to optimize performance of uncued stimuli.

### 4.7 Concluding remarks

Our study shows that self-reported fatigue in brain tumor patients is correlated with the level of DMN inhibition associated with phasic alertness. These results suggest that phasic alertness DMN inhibition represents a neuronal correlate for fatigue. The current findings are an important step in the development of an objective neuronal biomarker for fatigue in both meningioma and glioma patients, and may also apply to other patients that suffer from fatigue. Our findings that self-reported fatigue is especially correlated with DMN deactivation but not with CEN activation provides a new insight into the neurophysiology of fatigue.

## CRediT authorship contribution statement

**M.J. de Dreu:** Conceptualization, Methodology, Investigation, Formal analysis, Writing - original draft, Visualization, Project administration. **I.T. Schouwenaars:** Conceptualization, Methodology, Investigation, Writing - review & editing, Project administration. **G.J.M. Rutten:** Conceptualization, Supervision, Writing - review & editing, Funding acquisition. **N.F. Ramsey:** Conceptualization, Methodology, Supervision, Writing - review & editing, Funding acquisition. **J.M. Jansma:** Conceptualization, Methodology, Software, Supervision, Writing - original draft, Writing - review & editing, Funding acquisition, Project administration.
